# Supplementary material for: “You always have a high sugar if you don’t communicate”: A qualitative secondary analysis of ‘Diabetes Together’ process evaluation data from couples in South Africa
Source: PLOS Glob Public Health. 2025 Feb 26;5(2):e0004089. doi: 10.1371/journal.pgph.0004089 (PMC11864531; doi:10.1371/journal.pgph.0004089)
Supplement: S3 Appendix — Quotes from intervention-naïve participants. (DOCX) [file pgph.0004089.s003.docx]

**S3 Appendix: Quotes from intervention-naïve participants**

| **Theme** | **Quote** | **Person** |
| --- | --- | --- |
| Tone of discussions | *I get irritated sometime because I feel that he is asking me nonsense […] sometimes he knows that there are things that are caused by him bothering me here at home. Then that causes my sugar levels to escalate to a very high level you see.* | Female PLWD |
| Tone of discussions | *There are some moods with diabetes. I think when their sugar is high they get cranky* | Female partner |
| Tone of discussions | *I don’t know if this diabetic condition causes him to have these mood swings, or the temper thing, or is it, like a man thing that is the problem, I don’t know. Whichever way it is, I really don’t know, but he is different, you know. And the older he gets, the worse it gets.* | Female partner |
| Listening | *He does listen, he lends his ears […] but it goes in the one ear and goes out the other one […] I spoke to him [about diabetes] until I also even gave up […] he takes [diabetes] very lightly* | Female PLWD |
| Openness | *It is all about sharing. Talking to each other, about how he feels, and give each other support, and […] as I said to him, openness will help us. If you feel something, you have to be open, so we can get help.* | Female partner |
| Openness | *if there is something that she does which I don’t like I will tell her. The same thing she will tell me that kind of thing […] we are very open.* | Male PLWD |
| Openness | *we don’t have fear because we talk about everything. And we share, because [there is no] shame to be a diabetic, it can happen to anybody […] So we talk about it but I don’t [think] there is a fear because if there was fear obviously we would have talked about it […] we are open to any challenge that comes along our way.* | Female partner |
| Being informed | *[my partner’s] only fear was about the baby because I was strictly breastfeeding. So, I told him that there is nothing wrong with breastfeeding while living with diabetes […] He was thinking that when the child is breast fed it is easy for the illnesses to be passed on to the baby through breast milk […] I told him that nothing can happen, but he still has some doubts because he is not hundred percent sure.* | Female PLWD |
| Being informed | *I make sure she eats at the right time because sometimes she says she is hungry, feels dizzy and wants to collapse […] She gets angry at times and we argue, but then I understand her. I am ok.* | Male partner |
