# Supplementary material for: “You always have a high sugar if you don’t communicate”: A qualitative secondary analysis of ‘Diabetes Together’ process evaluation data from couples in South Africa
Source: PLOS Glob Public Health. 2025 Feb 26;5(2):e0004089. doi: 10.1371/journal.pgph.0004089 (PMC11864531; doi:10.1371/journal.pgph.0004089)
Supplement: Attachment — Submitted filename: Communication paper - cover letter REVISIONS.docx [file pgph.0004089.s006.docx]

Dear PLOS Global Public Health Editors and Reviewers

Thank you for taking the time to read and review our submission ‘“You always have a high sugar if you don’t communicate”: a qualitative secondary analysis of ‘Diabetes Together’ process evaluation data from couples in South Africa’.

We have addressed editors’ and reviewers’ comments with a point-by-point response below and the reviewed manuscript includes tracked changes.

***Editorial comment: Please include a complete copy of PLOS’ questionnaire on inclusivity in global research in your revised manuscript***

We have completed a copy of the questionnaire on inclusivity, added a reference to it in the methods section (page 6) and submitted it as Supplementary Appendix 2.

***Editorial comment: Please review your reference list to ensure that it is complete and correct***

We have reviewed our reference list and believe it is complete and correct.

***Reviewer 2: Title should be more attractive and smart***

We feel our title is attractive because it includes a quote from a study participant illustrating a connection between communication and type 2 diabetes. If there is a specific recommendation for change from the editorial board or reviewers, we are happy to consider it.

***Reviewer 2: Is Food habits of T2D patients considered in this study?***

While this paper specifically focused on the communication between partners in the context of the T2D management, the intervention included an educational session on the importance of healthy eating for managing diabetes (see the workshop agenda in Table 1, page 6). The aim of this section was to change the food habits of people living with diabetes and to inform partners about what healthy food habits will help with managing diabetes. In Lynch et al. (2024) (reference number 10 in our submitted paper) our process evaluation identified that some participants reported improving their dietary habits following the intervention (see [here](https://www.tandfonline.com/doi/pdf/10.1080/17441692.2024.2386979), page 10).

***Reviewer 2: Sampling method mentioned need to be more details.***

We have added details about sampling to the manuscript (see page 8, lines 148-52) as follows: “PLWD were approached at primary care diabetes clinics or contacted proactively following previous involvement in diabetes management studies with the Chronic Diseases Initiative for Africa (CDIA). Convenience sampling was used to ensure a minimum number of couples were available and in recognition of the challenges of recruitment during COVID.”

***Reviewer 2:*** ***Is Qualitative Data Translating process follow the standard rules?***

We are unclear what rules the reviewer is referring to here. We asked for clarification but did not receive further guidance on this comment.

We have amended the data analysis section (page 11) to clarify that we followed Braun and Clarke’s (2006)^[1]^ 6 stages to thematic analysis. We included the Standards for Reporting Qualitative Research in appendix S1 (referenced on page 6) to provide transparency on our approach to this qualitative study.

We have expanded the section about interpretation and translation (page 9) to clarify how non-English statements made during focus group discussions and interviews were translated and how interpretation was quality controlled to ensure accuracy.

***Conclusion***

We believe the changes have strengthened our paper and look forward to your response to our resubmission.

Yours faithfully

L Lynch

Lucy Lynch, Public Health Registrar

School of Primary Care, Population Sciences and Medical Education; Faculty of Medicine

University of Southampton

AC20, Level C, South Academic Block, University Hospital Southampton, Southampton SO16 6YD

1. Braun, V., & Clarke, V. (2006). Using thematic analysis in psychology. Qualitative Research in Psychology, 3(2), 77–101. https://doi.org/10.1191/1478088706qp063oa
